# Cardiometabolic Alterations in the Interplay of COVID-19 and Diabetes: Current Knowledge and Future Avenues

**DOI:** 10.3390/ijms222212311

**Published:** 2021-11-15

**Authors:** Ali A. Rizvi, Andrej Janez, Manfredi Rizzo

**Affiliations:** 1 Department of Medicine, University of Central Florida College of Medicine, Orlando, FL 32827, USA; ali.rizvi@ucf.edu; 2 Division of Endocrinology, Diabetes and Metabolism, Department of Medicine, University of South Carolina, Columbia, SC 29208, USA; 3 Department of Endocrinology, Diabetes and Metabolic Diseases, University Clinical Center Ljubljana, 1000 Ljubljana, Slovenia; andrej.janez@kclj.si; 4 Department of Health Promotion Sciences Maternal and Infantile Care, Internal Medicine and Medical Specialties (Promise), School of Medicine, University of Palermo, 90100 Palermo, Italy

The severe acute respiratory syndrome coronavirus 2 (SARS-CoV-2) (coronavirus disease 2019 [COVID-19]) pandemic has raged for almost two years, with few signs of a sustained abatement or remission [1]. It has affected not only the daily lives of most of the global population but has also affected the delivery of medical care to our patients [2]. It is evident from epidemiologic indicate that a major contributor to the severity of COVID-19 and its complications remains the presence or emergence of diabetes in the affected individuals [3,4]. In a converse fashion, COVID-19 has had a devastating impact on the health of patients with diabetes. More than a third of people with COVID-19 who require hospitalization suffer from diabetes, with a case fatality rate of approximately 25% [5]. Even after adjustment for sociodemographic factors and comorbid conditions, the risk of severe morbidity and mortality is up to 250% higher among people with diabetes, with overall mortality rates being 50% higher than pre-COVID times [6].

Factors associated with poor outcomes include comorbid conditions such as coronary artery disease, congestive heart failure, and chronic kidney disease. as well as the presence of concurrent obesity, metabolic syndrome, and poor glucose control [7,8]. Speculation on the potential pathways underlying the bidirectional and mutually deleterious relationship between glucometabolic perturbations and COVID-19 have encompassed the disruption of glucose metabolism, immune modulation, and coagulation and inflammatory responses, as well as exacerbation of endothelial dysfunction by acute hyperglycemia and the proinflammatory state [9]. Indeed, it is known that endothelial dysfunction is a strong predictor of future cardiovascular events [10], and some authors have even suggested that COVID-19 is, in the end, an endothelial disease [11].

At molecular level, the dysfunction of vascular endothelium is a key factor for the formation and progression of atherosclerosis, where there is a close interplay between enhanced inflammation, endothelial dysfunction and pro-atherogenic lipid alterations [12]. Indeed, low-density lipoproteins (LDL) with smaller size and more density have greater atherogenicity then larger counterparts due to several specific physico-chemical and metabolic properties, including prolonged residency time in plasma because of reduced affinity to LDL receptor, greater entry and retention into the arteries as well as enhanced oxidative susceptibility [13]. Several authors have emphasized that obesity-related and diabetes-related inflammation and endothelial dysfunction have a crucial impact on disease severity in COVID-19 patients [14,15] and, very interestingly, it has been recently shown that low concentrations of high-density lipoproteins (HDL) together with elevated levels of triglycerides predict COVID-19 severity [16]. These two lipid altertations are usually accompanied by the predominance of small, dense LDL, constituting the so-called lipid triad or atherogenic lipoprotein phenotype [17], a lipid trait that is very common in patients at high cardiovascular risk and more evident is some ethnicities [18,19,20,21,22].

In addition, in patients with diabetes or obesity endothelial inflammation, and atherosclerosis are closely linked to alterations in cytokine biomarkers [23], which are also strictly associated with atherogenic small, dense LDL [24]; therefore, concomitant molecular alterations in obesity and diabetes, including those in inflammatory adipokines and atherogenic lipoproteins enhance their overall cardiometabolic risk. Investigating such molecular mechanisms at the basis of atherosclerosis formation and progression can help to assess better strategies for proper treatment; for instance, the concomitant management of multiple lipid alterations is able to reduce the risk for cardiovascular events and the rates of progression of atherosclerotic disease [25]. This is particularly true during the current pandemic where we have observed a significant increase in cardiovascular complications in patients with diabetes or obesity [26,27]. In this context, some novel antidiabetic agents, such as glucagon-like peptide (GLP)-1 receptor agonists seems to play a role due to their anti-inflammatory and anti-atherogenic/thrombotic effects [28,29], with a likely direct mechanism against COVID-19 onset and severity [30]. Notably, these novel drugs are also able to reduce small, dense LDL [31], in contrast to what found with the use of some traditional antidiabetic drugs [32].

A coordinated response to the continuing pandemic will benefit from a careful analysis of the diabetes- and obesity-related risks of exposure, infection, and hospitalization. Notably, the indirect impact of the pandemic’s effects on chronic disease management, delivery of health services, modifications in health behaviors, and rapid attention to acute complications has not been well quantified. Patients with COVID-19 experience deterioration of cardiovascular and renal systems, both of which are causes of serious complications in presence of diabetes and obesity [8,33]; treatments must be monitored very carefully, keeping in mind that their adhearance and duration is critical for reaching the theraputical goal [34]. Very recently, an international panel of experts have emphasized that up to 50% of people who have died from COVID-19 had metabolic and vascular disorders; therefore, patients with diabetes and obesity need to be carefully managed and properly treated, now more than ever [35].

With this Special Issue, we aim to provide an update to the scientific and medical community about the close relationships between COVID-19 and diabetes, obesity, and cardiometabolic disorders. We wish to solicite articles expounding on the implications of the current pandemic on the population with diabetes and its COVID-19–related morbidity and mortality, investigating the predictors of severe adverse outcomes, and highlighting the importance of epidemiologic studies to identify the needs for the next phase of the pandemic. Therefore, we welcome the submission of high-quality manuscripts dealing with the current knowledge on the molecular mechanisms and disease processes in order to enhance current understanding and provide perspectives for the future. Papers addressing the basic sciences and pathophysiology, rather than clinical aspects, are therefore encouraged and preferred. We sincerely hope that the resulting compendium of state-of-the-art publications will lead to increased understanding and pave the translational paths to improved patient care this critical phase of the global COVID-19 pandemic.
